# A primer on tRNAs, tRNA-m^1^A58 modification, and tRNA derivatives in T-cell activation

**DOI:** 10.3389/fcell.2025.1683213

**Published:** 2026-01-06

**Authors:** Chuanxiang Zhao, Juxiang Shen

**Affiliations:** Institute of Medical Genetics and Reproductive Immunity, School of Medical Science and Laboratory Medicine, Jiangsu College of Nursing, Huai’an, China

**Keywords:** T cells, tRNA, tRNA-m1A58 modification, tRNA derivatives, tRFs

## Abstract

Upon antigen recognition, naive CD4^+^ T cells are activated, exiting quiescence to undergo rapid activation, clonal expansion, and differentiation into effector functions against pathogens. T-cell activation and clonal expansion necessitate the biosynthesis of millions of new protein copies. Recent technological advancements in small RNA sequencing have revealed a highly complex and dynamic repertoire of cellular tRNAs, tRNA-m^1^A58 modification, and tRNA derivatives during T-cell activation. This review outlines the basic framework of the biogenesis and biological functions of tRNAs, tRNA-m^1^A58 modification, and tRNA derivatives. Importantly, we elucidate how m^1^A58 modification regulates translation through multilevel mechanisms involving initiation, elongation, and termination. Furthermore, this review provides a comprehensive overview of the dynamic changes in tRNA expression repertoires and the impacts of tRNA-m^1^A58 modification and tRNA derivatives on T-cell activation. This review aims to offer novel insights into the molecular mechanisms underlying T-cell activation, facilitating the development of more effective therapeutic strategies for treating T-cell-related diseases.

## 1 Introduction

T lymphocytes are crucial components of the immune system and play a significant role in the adaptive cellular immune response. T cells originate from multipotent hematopoietic stem cells in the bone marrow and mature in the thymus. Mature naïve T cells subsequently migrate into peripheral immune organs where they are activated by antigens, eventually differentiating into distinct effector T-cell subpopulations or memory T cells (Lan et al., 2024). To provide the building blocks required for cell growth and proliferation, T lymphocytes rapidly translate millions of copies of new proteins in a short time window through transcriptional, posttranscriptional, and translational mechanisms. A principal mechanism involves augmenting RNA output at the transcriptional level, consequently driving protein production (Gao et al., 2024). Therefore, there is an urgent need to identify dynamic changes and the functional roles of RNA in T-cell activation and proliferation, which points to a previously unidentified therapeutic target to alleviate various T-cell-related inflammatory diseases and a new strategy for cancer immunotherapy.

As essential constituents of the translation apparatus, transfer RNAs (tRNAs) are conventionally viewed to modulate translation through their structural configurations and precise anticodon‒codon interactions with complementary mRNA sequences, delivering amino acids to the ribosome for protein synthesis (Marchingo, 2022). tRNA research is reemerging, as evidenced by documentation of tRNA engagement in diverse pathways beyond protein synthesis. Their regulation is closely related to diverse tRNA chemical modifications and tRNA derivatives (tRNA-derived small RNAs, tsRNAs), which leverage the preexisting mRNA pool (Malik et al., 2014; Peschek and Tuorto, 2025). Mammalian tRNAs, as the most extensively modified RNA molecules in cells, contain an average of 14 modified nucleotides per molecule (Boccaletto et al., 2018). tRNA modifications achieve precise and efficient regulation of protein synthesis by modulating tRNA structure maturation and stability, codon decoding accuracy, translation elongation rates, selective mRNA translation, m^6^A-dependent mRNA decay, and recruitment of translation initiation complexes (Malik et al., 2014; Pan, 2018; Linder et al., 2025). Notably, differences in tRNA expression patterns enable proliferating and differentiating cells to exhibit distinct translational programs (Gingold et al., 2014). In recent years, substantial effort has demonstrated the involvement of tRNA, tRNA modifications and tRNA derivatives in T-cell activation and proliferation and in the adaptation of cellular functions to changing environments by regulating the translation machinery, thereby modulating immune responses.

In this review, we systematically summarize the current knowledge concerning the biogenesis and biological functions of tRNAs, tRNA-m^1^A58 modification and tRNA derivatives. We further summarize emerging insights into their critical roles in T-cell activation (Table 1). On the basis of existing evidence, we additionally highlight the challenges and future directions in this field. A deeper mechanistic understanding of the tRNA epitranscriptome may provide novel therapeutic strategies for treating T-cell-associated immune disorders and malignancies.

**TABLE 1:** The effect of tRNA modification/derivative on T cell.

tRNA modification/derivative	Position	Effect on T cell	Reference
Wybutosine and ms2t6A	At anticodon position 37 of tRNAᴸʸˢ (UUU) and tRNAᴾʰ^e^ (GAA)	The decreased level at 20 h post-activation	Rak et al. (2021)
tRNA-m^1^A58 modification	At position 58 of the TψC loop in mature tRNAs	Promoting the translation elongation of Myc mRNA for rapid T cell expansion	Liu et al. (2022)
		Diminishing TH1 and TH17 cell differentiation by TRMT61A deficiency *in vitro*	Liu et al. (2022)
		The opposite Treg cell differentiation by TRMT61A deficiency *in vitro* versus *in vivo*	Liu et al. (2022), Miao et al. (2025)
		Regulating mitochondrial protein synthesis by TRMT61B-mediated mitochondrial tRNA m1A58 modification?	Richter et al. (2018)
		Impairing CD8^+^ T cells tumor-killing function by disrupting TRMT61A deficiency-induced cholesterol biosynthesis	Miao et al. (2025)
SLFN2 deficiency-induced tiRNAs	—	Suppressing IL-2 receptor translation	Yue et al. (2021)
Specific tRFs in extracellular vesicles that carry RNA cargo	—	Inhibiting IL-2 production	Chiou et al. (2018)
tRF3009 derived from tRNA-Leu-TAA	—	Inducing oxidative phosphorylation	Geng et al. (2021)

## 2 tRNA, tRNA m^1^A58 modification and tRNA derivatives

### 2.1 Biogenesis and biological functions of tRNA

**FIGURE 1:**
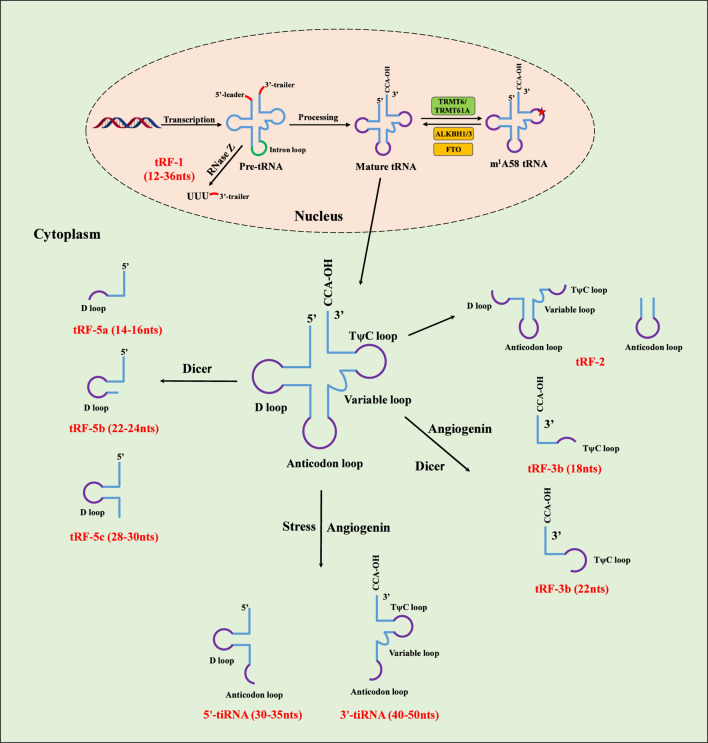
Biogenesis of tRNA, tRNA m^1^A58 methylation and tRNA derivatives. In the nucleus, pre-tRNAs are transcribed from tRNA genes by RNA polymerase III and then processed by ribonucleases to produce mature tRNA. Cytosolic tRNA m^1^A58 is introduced by the methyltransferases TRMT6 and TRMT61A. The demethylases responsible for m^1^A58 demethylation include ALKBH family members (ALKBH1 for cytoplasmic and mitochondrial tRNA modification and ALKBH3 for cytoplasmic tRNA modification) and fat mass and obesity-associated proteins (FTOs for cytoplasmic tRNA modification). tRNA m1A58 modification regulates translation by promoting the maturation and stability of tRNA^iMet^ and eEF1-tRNA binding and influencing ribosomal stalling. tRF-1 is generated from pre-tRNA and cleaved by RNase Z or ELAC2 at the 3′trailer in the nucleus. tRF-2s are derived from the anticodon loop of mature tRNA under hypoxic conditions. tRF-3s are derived from the cleavage of the TψC loop by angiogenin, Dicer, or members of the ribonuclease A superfamily at the mature tRNA. tRF-5s are decomposed from the 5ʹ end of the tRNA around the D-loop by Dicer cleavage. Under stress induction, 5′-tiRNAs and 3′-tiRNAs are cleaved at the anticodon loop of mature tRNA by angiogenin.

In the human genome, there are more than 400 tRNA genes, more than 200 of which are usually expressed in each cell and some of which exhibit cell- or tissue-specific expression patterns (Schimmel, 2017). In the nucleus, RNA polymerase III (Pol III) catalyzes the transcription of functional tRNA genes to generate primary products, known as precursor tRNAs (pre-tRNAs) (Phizicky and Hopper, 2010). Pre-tRNAs must subsequently undergo posttranscriptional processing, including ribonuclease P-mediated trimming of the 5′-leader and RNase Z-mediated cleavage of 3′-trailer sequences, addition of the CCA sequence at the 3′-end, intron splicing from their respective ends and base modifications (Abelson et al., 1998; Phizicky and Hopper, 2010). The translation-proficient mature tRNAs are subsequently formed and then exported to the cytosol. In the cytoplasm, mature tRNAs, with lengths ranging from 75 to 93 nucleotides, feature a typical “clover” secondary structure comprising a D-loop, an anticodon loop, a variable loop, a TψC-loop and an acceptor arm, as well as an L-shaped tertiary structure maintained by hydrogen bonds (Schimmel, 1995) (Figure 1). In addition to traditional functions that affect translation via their structures and interactions with the corresponding mRNA codons, specific mature tRNAs, especially select arginine tRNAs, can promote mRNA degradation by recruiting the CCR4-NOT complex to the P-site of translating ribosomes (Zhu et al., 2024).

### 2.2 Biogenesis and biological functions of tRNA m^1^A58 modification

During posttranscriptional processing, methylation is one of the most prevalent types of tRNA modifications; it is critical for stability, maturation, and function and intimately associated with tRNA roles as adapters in protein synthesis (Wu et al., 2024). The RNA modification databases document approximately 22 distinct methylation types in human cytoplasmic tRNAs and 8 types in mitochondrial tRNAs (Boccaletto et al., 2022). The principal types of tRNA methylation include N5-methylcytosine (m^5^C), N1-methyladenosine (m^1^A), N7-methylguanosine (m^7^G), N1-methylguanosine (m^1^G) and N5-methyluridine (m^5^U), all of which can be detected and quantified via high-throughput sequencing (Wu et al., 2024). Methylated tRNA^iMet^ tends to be preferentially engaged by ribosomes for translation initiation and elongation (Liu et al., 2016). Furthermore, methylation within the tRNA anticodon loop is critical for ensuring rapid and accurate mRNA decoding, primarily by controlling the degradation of functionally associated m^6^A-modified mRNA codons and enhancing ribosomal A-site occupancy, thereby modulating the translation efficiency of corresponding codons (Linder et al., 2025). In this context, m^1^A methylation has attracted increased attention.

m^1^A methylation is predominantly localized on the nitrogen at position 1 of various adenosine sites throughout the tRNA molecule, comprising the D-loop and T-loop (Oerum et al., 2017). This modification brings a positive charge on this atom, and thus is highly likely to play regulatory functions. Cytosolic tRNA features m^1^A modification at five distinct positions (9, 14, 22, 57, and 58), with positions 9 and 58 additionally present in mitochondrial tRNA (Oerum et al., 2017). The precise sites of m^1^A modification depend on the tRNA species and organism (Smoczynski et al., 2024). Recently, the highly conserved intron of tRNA-tyrosine was identified as essential for m^1^A58 installation in the T-loop of mature tRNAs (Catherine et al., 2025). m^1^A58 methylation is dynamically regulated by key enzymatic regulators, namely, methyltransferases (writers) and demethylases (erasers), that respond to cellular demands, environmental stress, and pathological conditions (Figure 1). Cytosolic tRNA m^1^A58 is introduced by the methyltransferases TRMT6 and TRMT61A, whereas human mitochondrial tRNA m^1^A58 is modified by TRMT61B (Chujo and Suzuki, 2012; Safra et al., 2017). The demethylases responsible for m^1^A58 demethylation include ALKBH family members (ALKBH1 for cytoplasmic and mitochondrial tRNA modification and ALKBH3 for cytoplasmic tRNA modification) and fat mass and obesity-associated protein (FTO for cytoplasmic tRNA modification) (Kawarada et al., 2017; Wei et al., 2018; Chen et al., 2019). Concurrently, ALKBH3 and FTO additionally mediate m^6^A demethylation in mRNAs (Wei et al., 2018; Tu et al., 2025). The dual functions of ALKBH3 and FTO in tRNA m^1^A58 and m^6^A-modified mRNAs collectively introduce considerable complexity into cellular homeostasis and cellular functional adaptation to fluctuating environments.

With the advances in next-generation sequencing (NGS) technologies, many experimental methods have been designed to profile RNA modifications. Here we briefly introduce characteristics of current methods to study m^1^A58 modification in Table 2.

**TABLE 2:** Tools to study m^1^A58 modification.

Category	Techniques	Purpose	Advantages	Limitations	Refs
Modification detection and quantification	Liquid Chromatography-Mass Spectrometry (LC-MS)	Absolute quantification of nucleosides after enzymatic digestion; measures global m^1^A58 modification levels	Providing absolute quantification with high sensitivity and accuracy	Loss of sequence context for individual tRNA molecules	Zhang et al. (2023)
	m^1^A-MAP-seq	A single-base resolution to identify m^1^A modification	Excellent readthrough efficiency and relatively high mutation frequency	The sequence context of RNA affect the mutation rate	Li et al. (2017)
	Primer-extension	Validate the new detection technique	Precise modification position	RNA targets of high abundance and existing sequence knowledge	Chengqi Yi (2011)
	Nanopore Direct RNA Sequencing	Directly reads native RNA molecules, detecting m^1^A58 and co-modifications in real-time via changes in ionic current	Preservation of modification information, long read length and high resolution, and accurate quantification of low-abundance transcripts and modification sites	High computational demand, relatively lower throughput and higher cost	Lucas et al. (2023)
Functional mechanism investigation	CRISPR/Cas9 Screening	Systematically knocking down modifying enzymes to assess genomic impacts	Enabling unbiased phenotypic discovery and revealing biological roles	Limited model applicability and temporal resolution	Zhang X. et al. (2025)
	Cre-Lox system			Off-target effects	Song and Palmiter (2018)
Clinical/translational exploration	Differential Expression and Correlation Analysis	Correlate expression of modifying enzymes and tRF sequencing data with clinicopathological features in disease cohorts	Establishing clinical relevance and identifying potential biomarkers	Lack of causal inference and the results potentially for confounding by other variables	Shi et al. (2025)

### 2.3 Biogenesis and biological functions of tRNA derivatives

tRNA-derived fragments (tRFs) and tRNA halves (tiRNAs) constitute discrete classes of small noncoding RNAs derived from precursor or mature tRNAs through tightly and finely regulated processing (Zhang B. et al., 2025). On the basis of their origins and cleavage sites, tRFs (approximately 14–30 nts), which originate from mature or precursor tRNAs, are categorized into tRF-1, tRF-2, tRF-3, tRF-5, and i-tRF. Specifically, tRF-3 is cleaved by angiogenin (ANG), Dicer, or members of the ribonuclease A superfamily at the TψC loop of mature tRNA and comprises two subtypes, tRF-3a (18 nts) and tRF-3b (22 nts). Therefore, m^1^A58 modification may regulate the biogenesis of tRF-3.

tiRNAs (29–50 nts) are generated via ANG-mediated specific cleavage at the mature tRNA anticodon loop under stress stimuli and are further subclassified into 5′tiRNAs (30–35 nts) and 3′tiRNAs (40–50 nts) (Zhang B. et al., 2025) (Figure 1). tRFs and tiRNAs, initially regarded as degradation byproducts, are now established through accumulating evidence as regulators of intracellular gene transcription, protein translation, and epigenetic modulation (Zhang B. et al., 2025). Functionally, tRFs engage in diverse molecular mechanisms, including miRNA-like target gene silencing, RNA processing and degradation, histone modification, ribosome assembly, and the unfolded protein response (Zhang B. et al., 2025). Specifically, ALKBH3-induced 5′-tRF-GlyGCC strengthened ribosome assembly via direct interaction with the 40S ribosome and prevented cancer apoptosis through interaction with Cyt c (Chen et al., 2019; Wu et al., 2021).

## 3 The regulatory effects of m^1^A58 modification on tRNAs and tRFs

**FIGURE 2:**
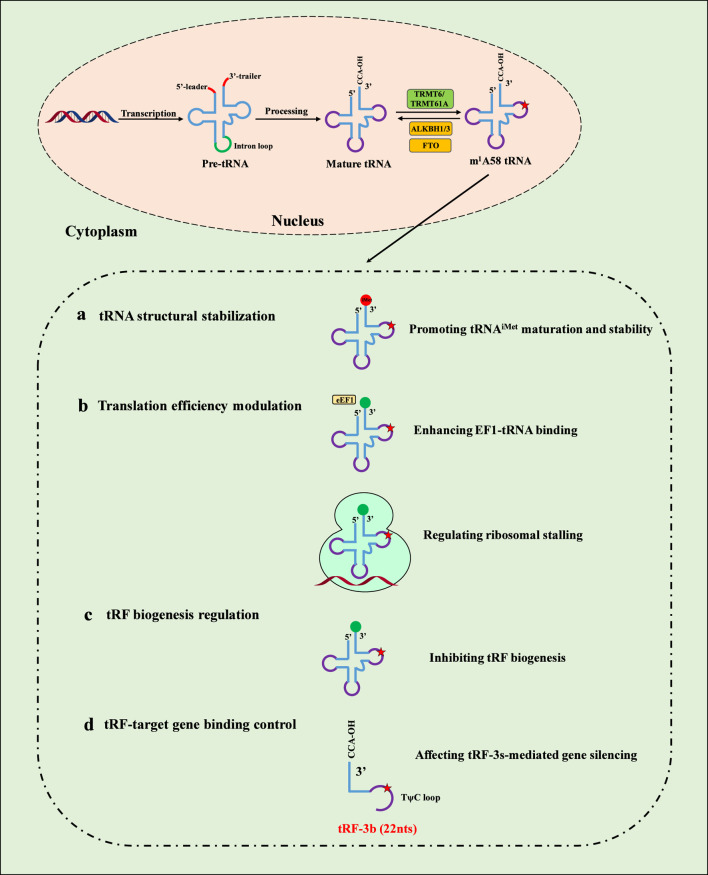
The regulatory effects of m^1^A58 modification on tRNA and tRFs. a, tRNA structural stabilization. m^1^A58 modification stabilizes the tRNA fold and facilitates proper maturation. b, Translation efficiency modulation. m^1^A58 modification enhances translation efficiency by promoting eEF1A-tRNA binding, thereby facilitating ribosomal translocation without significantly affecting tRNA abundance or aminoacylation. m^1^A58 modification acts as a translational checkpoint by modulating ribosomal stalling at specific codons and regulating ribosome-mRNA interactions. c, tRF biogenesis regulation. m^1^A58 modification suppresses tRF biogenesis. d, tRF-target gene binding control. m^1^A58 methylation attenuates tRF-3b-mediated argonaute (Ago)-dependent gene silencing.

The m^1^A58 modification, a conserved post-transcriptional alteration present in cytosolic and mitochondrial tRNAs across bacteria, archaea, and mammals, plays multifaceted roles in regulating tRNA functionality and downstream processes (Saikia et al., 2010). Its regulatory effects can be systematically categorized into four distinct aspects: (a) tRNA structural stabilization, (b) translation efficiency modulation, (c) tRFs biogenesis regulation, and (d) tRF-target gene binding control (Figure 2).

### 3.1 tRNA structural stabilization

The m^1^A58 modification is essential for maintaining the structural thermostability of tRNAs, particularly exemplified by the initiator tRNA-Met (tRNA^iMet^). This modification stabilizes the tRNA fold and facilitates proper maturation, thereby preventing degradation and ensuring functional integrity (Horie et al., 1985; Anderson et al., 1998; Tang et al., 2020; Yared et al., 2023; Tao et al., 2025). For instance, deficiency in m^1^A58 leads to tRNA^iMet^ destabilization and subsequent degradation, underscoring its critical role in structural maintenance.

### 3.2 Translation efficiency modulation

m^1^A58 exerts broad influence on translation through mechanisms involving initiation, elongation, and termination. It is indispensable for translation initiation, as m^1^A58 deficiency impairs this process by compromising tRNA^iMet^ stability (Liu et al., 2016). During elongation, m^1^A58 enhances translation efficiency by promoting eEF1A-tRNA binding, thereby facilitating ribosomal translocation without significantly affecting tRNA abundance or aminoacylation (He et al., 2024). Additionally, TRMT6-mediated m^1^A58 modification acts as a translational checkpoint by modulating ribosomal stalling at specific codons and regulating ribosome-mRNA interactions (Tao et al., 2025). In the context of viral replication, m^1^A58 in human tRNA^Lys,3^, together with m5U54, partially serves as a stop signal due to systematic miscoding, thereby ensuring efficacy and fidelity during HIV-1 reverse transcription (Auxilien et al., 1999; Fukuda et al., 2021). The incorporation pathways of m^1^A58 further highlight its regulatory specificity; for example, in yeast, elongator tRNAs follow a sequential modification circuit (Ψ55→m5U54 → m^1^A58), whereas initiator tRNAiMet modification occurs independently of prior alterations (Yared et al., 2023; Smoczynski et al., 2024).

### 3.3 tRF biogenesis regulation

Although less extensively characterized, m1A58 modification contributes to the regulation of tRNA-derived fragment biogenesis. Theoretically, tRNA methylation enhances their stability, thereby suppressing tRF biogenesis; consequently, tRFs are derived from hypomethylated tRNAs and exhibit hypomethylation. For example, ALKBH1 and ALKBH3 participate in a stress-specific process that destabilizes tRNAs and facilitates tRNA cleavage to generate tRNA derivatives around anticodon regions (Chen et al., 2019; Rashad et al., 2020). Nevertheless, after ALKBH1 manipulation and stress exposure, tRNA cleavage exhibits nonuniform patterns across different tRNA species in terms of cleavage sites (Rashad et al., 2020). Notably, single-read tRNA-seq analyses revealed the functional crosstalk of m^1^A58 with other tRNA modifications in the human tRNAome to regulate tRF biogenesis (Hernandez-Alias et al., 2023). Recently, tRF-3b enriched with m^1^A58 modification was revealed, which affects gene silencing by tRF-3s (Su et al., 2022). Hence, tRNA m^1^A58 methylation governs the biogenesis of tRNA derivatives on the basis of the specific tRNA, cleavage location, and coexistence of other modifications.

### 3.4 tRF-target gene binding control

The presence of m^1^A58 in tRNAs can indirectly affect tRF-mediated gene silencing or binding interactions. tRFs originating from m^1^A58-modified tRNAs may exhibit altered binding affinities to target mRNAs or regulatory proteins, thereby fine-tuning post-transcriptional gene expression networks. Recently, the seed region of 22-nt tRF-3bs was found to be highly enriched for TRMT6/61A-dependent m^1^A58 methylation, which attenuates tRF-3b-mediated argonaute (Ago)-dependent gene silencing involved in the unfolded protein response (Su et al., 2022). The detailed mechanism stems from impaired target mRNA base pairing rather than compromised AGO association with tRF-3bs (Su et al., 2022). However, the manner in which m^1^A58 methylation affects the generation and function of tiRNAs remains incompletely characterized. This aspect warrants further investigation to elucidate precise mechanisms.

In summary, m^1^A58 modification orchestrates tRNA function and translation through multilevel mechanisms, including structural stabilization and efficiency modulation, while also impinging on tRF biogenesis and target interactions. Dysregulation of m^1^A58 methylation is associated with pathological conditions such as cancer, neurological disorders, and metabolic abnormalities, highlighting its broad physiological impact (Suzuki, 2021; Wu et al., 2024).

## 4 The biological functions of tRNA, tRNA m^1^A58 methylation and tRNA derivatives during T-cell activation

### 4.1 Dynamic changes in the tRNA expression profile during T-cell activation

As the primary effector cells for defending the host against pathogens, naive CD4^+^ T cells are activated upon antigen recognition, exiting quiescence to undergo rapid activation, massive clonal expansion, and differentiation into effector cells (Ryan D Michalek, 2010; An et al., 2025). During these processes, they undergo sequential reprogramming through distinct cellular stages with substantial bioenergetic and biosynthetic demands, including early signaling activation (0–6 h), metabolic reprogramming (6–12 h), pre-cell cycling (12–24 h) and proliferation (24–72 h) (Ryan D Michalek, 2010; Hidehiro Yamane, 2013). The decoding of genetic information in the tRNA pool during T-cell activation is crucial for fundamentally massive translation of millions of protein copies within constrained timeframes to drive growth and proliferation.

**FIGURE 3:**
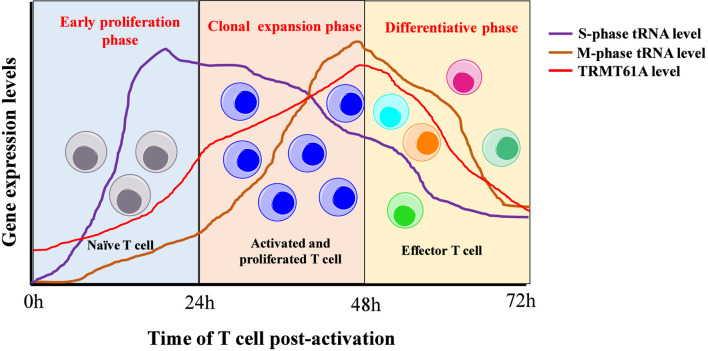
Dynamic changes of TRMT61A and cell cycle phase-expression-related tRNA levels during T-cell activation.

Upon T-cell antigenic activation, tRNA and mRNA pools undergo dynamic, coordinated, and complementary changes to accommodate the altered codon usage requirements of proliferation-associated genes. During the early proliferation phase of the response, the expression of tRNAs encoding AT-ending codons is upregulated following anti-CD3/CD28 stimulation for the specified durations (20 h and 48 h). At 20 h post-activation, the cells presented peak S-phase gene expression-related tRNA levels, whereas the peak expression of tRNAs linked to M-phase genes occurred at 48 h post-activation (Rak et al., 2021) (Figure 3). The study employed tRNA sequencing, which is a powerful tool to investigate transcriptome-wide changes in abundance. However, the quantitative accuracy of the method can be influence by technical biases. Nevertheless, the tRNA pools returned to baseline levels during the differentiation phase after 72 h of anti-CD3/CD28 stimulation, possibly preventing excessive proliferation. However, this finding based on population-level sequencing may mask underlying heterogeneity between emerging effector and memory T cell subsets, which could possess distinct translational programs. Interestingly, tRNAs decoding CG-ending codons demonstrate increased A-site occupancy at 48 h and 72 h after activation relative to that of naïve T cells (Rak et al., 2021). This finding warrants future investigation.

Further complexity is uncovered by the analysis of individual tRNA genes. Notably, several specific tRNA genes within tRNA families present pronounced changes in expression at different time points of T-cell activation, reflecting the codon usage bias of specific proteins. Two unique tRNAs, selenocysteine and initiator-methionine tRNA (tRNA^iMet^), were downregulated at all time points after T-cell activation. In three tRNA families, Ala-AGC, Ala-TGC, and Val-AAC, the increase in anticodon-specific tRNA levels in activated cells stems from the upregulation of one or two tRNA genes with low basal expression in naive cells (Rak et al., 2021). This granular insight depends critically on the resolution of the sequencing method to distinguish between highly similar tRNA isodecoders. The level of adaptation between the tRNA repertoire and transcriptomic codon usage can influence the abundance of translated proteins across temporal stages (Malik et al., 2012). This coordinative interplay represents an essential mechanism to sustain high-level expression of specific genes. The physiological significance of these specifically expressed tRNAs, and the precise mechanisms behind the observed ribosome occupancy, constitute compelling avenues for future research, ideally employing single-cell omics and direct mechanistic perturbations in primary T cells.

Furthermore, human cellular proliferation and differentiation are found to represent opposite expression patterns of tRNA pools, reflecting codon usage preferences of protein-coding genes expressed in different cell types (Gingold et al., 2014; Rak et al., 2021). This difference enables the translational machinery to be locked into the two programs of “proliferation” and “differentiation”. These distinct translation programs enable cells in a differentiated state to maintain stability and prevent abnormal proliferation or transformation. In this mode, even if transcriptional noise arises, causing aberrant expression of pro-proliferative genes in cells, such genes cannot be efficiently translated owing to alterations in the tRNA repertoire at this stage (Gingold et al., 2014). This preserves the direction of cell fate during differentiation. Given that T-cell activation and proliferation are closely followed by Th-cell subset differentiation, the dynamic changes in specific tRNA repertoires during T-cell differentiation deserve further investigation.

Additionally, the adaptability of cells to the microenvironment is governed by global tRNA codon fitness (Sivan Navon, 2011). In particular, the sequences of highly expressed genes have undergone codon optimization (Gingold et al., 2012). Studies have shown that when demand exceeds supply, particularly when highly expressed mRNA codons of the transcriptome fail to acquire corresponding tRNA levels, inefficient allocation of resources such as ribosomes and diminished cellular adaptability can occur (Grzegorz Kudla et al., 2009). Protein synthesis is necessary for T-cell adaptation to multiple pathological conditions. Therefore, further exploration is needed to uncover dynamic tRNA profile alterations in T cells under pathological conditions, including cancer, infection, and autoimmune diseases.

### 4.2 tRNA m^1^A58 methylation and T cell biology

#### 4.2.1 tRNA m^1^A58 methylation promotes activation and proliferation by regulating mitochondrial protein synthesis and Myc mRNA translation elongation

tRNA molecules are highly enriched with posttranscriptional RNA modifications, some of which can regulate stability, translation efficiency, decoding rate, and fidelity. Human tRNA modifications exhibit both tissue-specific and cell type-specific characteristics, with their levels and activities dynamically fluctuating in response to cellular stressors or environmental cues. The tRNA-m^1^A58 modification functions as a translational checkpoint that is critical for the rapid synthesis of specific key functional proteins that drive T-cell expansion (Liu et al., 2022). tRNA-m^1^A58 modification is introduced by TRMT6/TRMT61A. Upon T-cell activation, TCR signaling upregulated TRMT6 and TRMT61A expression by regulating c-JUN and FOSL2, resulting in m^1^A58 modification of early-expressed tRNAs within a very short timeframe (6 h post-activation) (Liu et al., 2022). Following 6–12 h of activation, T cells initiate metabolic reprogramming to promote their rapid proliferation. In line with this, TRMT61A-mediated tRNA m^1^A installation enables efficient translation of ATP-citrate lyase (ACLY), a key protein required for cholesterol biosynthesis (Miao et al., 2025). Therefore, TRMT61A deficiency in CD8^+^ T cells impairs their tumor-killing function by disrupting cholesterol biosynthesis (Miao et al., 2025). Furthermore, T cells rely on functional mitochondria to fine-tune metabolic programming to address the bioenergetic demands of T cells in response to nutrient availability (Ron-Harel et al., 2016; Lareau et al., 2023). Consequently, dynamic shifts in the mitochondrial tRNA pool and its modification landscape precisely also regulate mitochondrial protein synthesis. The m^1^A58 modification in mitochondrial tRNAᴸʸˢ governs translation elongation and nascent chain stability, thereby fine-tuning mitochondrial protein synthesis (Richter et al., 2018). The mitochondrial tRNA^Lys^ m^1^A58 modification is catalyzed by TRMT61B (Chujo and Suzuki, 2012). Accordingly, evidence suggests that TRMT61B-mediated mitochondrial tRNA m^1^A58 modification plays an undeniable role in T-cell activation by regulating mitochondrial protein synthesis.

Although global tRNA m^1^A levels remain stable during early activation at the pre-cell cycle stage (12–24 h), TRMT61A-mediated m^1^A58 modification specifically elevates select tRNA species, facilitating robust translation of a set of critical genes (notably Myc), thereby promoting MYC-regulated cell cycling, mitosis, and metabolism (Liu et al., 2022; Pei and Kuchroo, 2022; Lin et al., 2023). TRMT61A deficiency does not impair signaling pathways such as the JAK-STAT5, IL-2, IL-7, ZAP70, and ERK pathways (Liu et al., 2022). This mechanism guides the naïve T-cell transition from quiescence to proliferation, accelerating post-activation expansion. tRNA-m^1^A58 modification promoted the translation elongation of Myc mRNA through the decoding of serine (AGC) and leucine (TTG/CTG) codons (Liu et al., 2022; Marchingo, 2022). This finding underscores the importance of m^1^A58 modification as a critical translational checkpoint that acts as a “gas pedal” for T-cell proliferation (Marchingo, 2022). Consequently, TRMT61A/TRMT6 ablation in T cells attenuates T-cell-mediated adoptive transfer-induced colitis (Liu et al., 2022).

Additionally, wybutosine and ms2t6A at anticodon position 37 of tRNAᴸʸˢ (UUU) and tRNAᴾʰ^e^ (GAA) were significantly reduced at 20 h post-stimulation, whereas most other modifications remained stable during T-cell activation (Rak et al., 2021). This critical discovery was enabled by tRNA-seq and liquid chromatography-mass spectrometry (LC-MS/MS), which underscores the necessity of a multi-methodological approach to capture a comprehensive epitranscriptomic landscape. However, the downstream m^1^A modifications of these two tRNAs remain unaltered. Notably, both tRNAs encode “slippery codons” predisposed to ribosomal frameshifting (Rak et al., 2021). Consequently, reduced wybutosine and ms2t6A modification levels accelerate translation, enabling cells to achieve faster protein synthesis, which is a critical requirement for rapid biomass accumulation during cellular proliferation, while compromising translation fidelity (Rak et al., 2021). This fidelity/frameshift regulatory mechanism in activated T cells may be exploited by HIV for replication. However, this mechanistic link was established not in primary T cells but in a HeLa cell model with a CRISPR/Cas9-mediated knockout of the wybutosine biosynthesis enzyme TYW1. While this reductionist system provides a clear proof-of-principle for the causal relationship between yW modification loss and increased frameshifting at the HIV gag-pol sequence, it also highlights a methodological gap; the translational fidelity network in this cancer cell line may not fully recapitulate the physiological context of activated primary T cells, where additional layers of regulation likely exist.

#### 4.2.2 tRNA-m^1^A58 methylation and T cell subsets: An unresolved association

In the case of thymic T-cell development under steady-state conditions, TRMT61A deficiency did not influence the process. However, the modification reduced activated T-cell proportions in the spleen and lymph nodes (Liu et al., 2022; Miao et al., 2025). With respect to T-cell differentiation, TRMT61A deficiency in naïve CD4^+^ T cells results in a diminished capacity to differentiate into Th1 and Th17 cells and induce Treg (iTreg) cells *in vitro* (Liu et al., 2022). Paradoxically, Trmt61a-deficient mice display an increased number of regulatory T (Treg) cells in mesenteric lymph nodes (Miao et al., 2025). Nevertheless, whether abnormal T-cell differentiation arising from TRMT61A deletion is indeed attributable to reductions in tRNA-m^1^A58 modification remains unknown.

### 4.3 tRNA derivatives inhibit T-cell activation

Upon T-cell receptor stimulation, metabolic processes are triggered to meet the energy demands for clonal expansion; however, intracellular reactive oxygen species (ROS) are concomitantly generated, inducing angiogenin-mediated tRNA derivative production (Emara et al., 2010; Franchina et al., 2018). The evidence highlights the suppressive effects of intracellular tRNA derivatives on T-cell activation and proliferation. Following stimulation with anti-CD3/CD28 for 12 and 24 h, Schlafen 2 (SLFN2) is upregulated in activated T cells and can directly bind to tRNAs, safeguarding them from oxidative stress-induced cleavage by ANG under high ROS levels (Yue et al., 2021). Consequently, elevated ROS trigger excessive tRNA cleavage into short tiRNAs (31–40 nts) in SLFN2 deficiency, subsequently suppressing IL-2 receptor translation (but not IL-2 production). As a result, SLFN2-deficient T cells fail to proliferate following T-cell receptor stimulation (Yue et al., 2021).

Additional evidence supports the suppressive effects of intracellular tRNA derivatives on T-cell activation, although the suppressive mechanisms exhibit nuanced distinctions. T-cell-activating signals promote multivesicular body (MVB) formation, consequently inducing MVB-mediated release of extracellular vehicles (EVs) abundant in specific tRFs derived from the 5′-end and 3′-internal regions of tRNAs lacking variable loops (Chiou et al., 2018). These tRFs can suppress T-cell activation and the synthesis of costimulatory cytokines, such as IL-2, which are essential for T-cell viability and functionality in response to stimulatory signals (Chiou et al., 2018). The divergent suppressive mechanisms of T-cell activation may be attributable to the type of tRNA derivatives or their origin from distinct tRNA species.

Furthermore, intracellular tRNA derivatives suppress T-cell activation by regulating energy metabolism. CD4^+^ T cells in lupus patients display 482 differentially expressed tRFs, among which tRF3009 derived from tRNA-Leu-TAA is induced by IFN-α and is positively correlated with the disease activity index and lupus nephritis (Geng et al., 2021). In turn, tRF-3009 has been shown to directly modulate IFN-α-induced oxidative phosphorylation (OXPHOS) metabolism in CD4^+^ T cells (Geng et al., 2021).

## 5 Conclusion

In summary, the tRNA epitranscriptome represent emerging and rapidly evolving frontiers in immunological research. To protect the host against pathogens, CD4^+^ T cells must undergo activation, massive clonal expansion, and differentiation into effector functions upon antigen recognition. These processes are accomplished through massive translation of new proteins within a short timeframe. With advancements in high-throughput sequencing, a highly complex and dynamic cellular tRNA epitranscriptome has been revealed during T-cell activation, shedding light on its regulatory roles in these processes.

Herein, we present a comprehensive summary of the biogenesis and functional roles of tRNAs, tRNA-m^1^A58 modification, and tRNA derivatives. More crucially, we provide current insights into how m^1^A58 modification governs translation through multilevel mechanisms involving initiation, elongation, and termination. Nevertheless, the influence of m^1^A58 methylation on the biogenesis and functional specificity of tRNA derivatives remains a critical knowledge gap.

Furthermore, this review provides a comprehensive overview of the dynamic changes in tRNA expression repertoires and the effects of tRNA-m^1^A58 modification and tRNA derivatives on T-cell activation. While tRNA derivatives are hypothesized to suppress T-cell activation, their underlying mechanisms-including potential roles in reducing functional tRNA pools, inhibiting translation machinery via stress granule formation, or exerting microRNA-like actions-remain elusive.

In summary, the tRNA epitranscriptome represent emerging and rapidly evolving frontiers in immunological research. Deeper mechanistic insights into their T-cell functions may enable novel immunotherapies for T-cell-related pathologies, including inflammatory disorders and malignancies. For example, small-molecule inhibitors targeting tRNA cleavage enzymes can be utilized to deplete suppressive tRNA derivatives, thereby enhancing the activity of tumor-infiltrating T cells. Conversely, m^1^A-modified tRNA analogs may be employed to rescue translation defects in inflammatory T cells.

## 6 Future perspectives

Building on the current understanding summarized above, several key directions warrant further investigation to advance our knowledge of tRNA-mediated regulation in T-cell biology:

First, addressing the critical gap in understanding how m^1^A58 modification influences tRNA derivatives is essential. This necessitates two interconnected lines of inquiry: (i) elucidating how the m^1^A58 mark, due to its spatial proximity to canonical tRNA cleavage sites, influences ribonuclease accessibility and cleavage specificity-thereby modulating tRF biogenesis-even in the absence of direct modification on the resulting tRF molecules; and (ii) determining whether m^1^A58-mediated alterations in tRF generation and sequence composition confer distinct functional properties on these fragments, ultimately shaping their roles in gene regulation and cellular signaling across different disease contexts. Furthermore, the structural uniqueness of tRNA introduces an additional layer of complexity: the potential cross-talk between m^1^A58 and other tRNA modifications, such as pseudouridine (Ψ). Future work should systematically evaluate how these modification networks collectively influence tRNA folding, ribonucleolytic susceptibility, and the functional output of tRFs.

Second, subsequent investigations should delineate the temporal dynamics of tRNA modifications through the implementation of advanced sequencing methodologies in the context of T-cell activation. We hypothesize that early T-cell activation involves the upregulation of specific tRNA isoforms and modifications to bolster translation of proliferation-related genes, whereas later stages may involve cleavage into tRFs to constrain excessive expansion. To rigorously validate this model, future work should employ direct RNA nanopore sequencing to quantitatively map the landscape of tRNA modifications across distinct T-cell states at high temporal resolution. This approach, combined with parallel tRF profiling, will precisely capture the coordination between modification dynamics and RNA derivative production under diverse physiological and pathological conditions.

Third, to unravel the subtype-specific roles of tRNA-m^1^A58 modification and tRNA derivatives across T helper cell subsets (e.g., Th1, Th17, and Tregs), it is essential to integrate multidimensional single-cell analyses. We propose performing single-cell RNA sequencing (scRNA-seq) on CD4^+^ T cells isolated from relevant disease models-such as rheumatoid arthritis (representing chronic inflammation) and melanoma (representing a tumor microenvironment)-across multiple time points post-activation (e.g., 0 h, 24 h, 72 h). This strategy will not only resolve transcriptional differences among subsets but also, when coupled with genetic or chemical perturbation of modifying enzymes like TRMT6/61A, infer changes in tRNA-modifying enzyme activity and link them to tRF biogenesis and functional specialization during T-cell differentiation.
